# Quality Criteria for Serious Games: Serious Part, Game Part, and Balance

**DOI:** 10.2196/19037

**Published:** 2020-07-24

**Authors:** Polona Caserman, Katrin Hoffmann, Philipp Müller, Marcel Schaub, Katharina Straßburg, Josef Wiemeyer, Regina Bruder, Stefan Göbel

**Affiliations:** 1 Multimedia Communications Lab Technical University of Darmstadt Darmstadt Germany; 2 Institute of Sport Science Technical University of Darmstadt Darmstadt Germany; 3 Research Group Didactics of Mathematics Technical University of Darmstadt Darmstadt Germany

**Keywords:** serious games, educational games, games for health, exergames, quality criteria, video games

## Abstract

Serious games are digital games that have an additional goal beyond entertainment. Recently, many studies have explored different quality criteria for serious games, including effectiveness and attractiveness. Unfortunately, the double mission of serious games, that is, simultaneous achievement of intended effects (serious part) and entertainment (game part), is not adequately considered in these studies. This paper aims to identify essential quality criteria for serious games. The fundamental goal of our research is to identify important factors of serious games and to adapt the existing principles and requirements from game-related literature to effective and attractive serious games. In addition to the review of the relevant literature, we also include workshop results. Furthermore, we analyzed and summarized 22 state-of-the-art serious games for education and health. The selected best-practice serious games either prove their effectiveness through scientific studies or by winning game awards. For the analysis of these games, we refer to “DIN SPEC 91380 Serious Games Metadata Format.” A summarized text states quality criteria for both the serious and the game part, and especially the balance between them. We provide guidelines for high-quality serious games drawn from literature analysis and in close cooperation with domain experts.

## Introduction

Serious games are digital games that not only entertain but also intend to achieve at least one additional goal, a so-called characterizing goal [1]. Serious games aim to accomplish this characterizing goal without compromising the experience of playing a game (player experience). Examples include educational games, such as Meister Cody – Talasia (Meister Cody GmbH) [2], and games for health, such as ExerCube (Sphery AG) [3] and Pokémon GO (Nintendo Co) [4]. They should, in general, motivate the player to achieve the characterizing goal through appropriate methods, an engaging player experience, and the use of appropriate interaction technology.

Serious games are not limited to training (exercising) or learning. They can also be used for other purposes and can be applied in almost every area. For example, America’s Army (US Army) [5] is used as a recruiting tool; Re-Mission (HopeLab) [6,7] aims to change attitude, knowledge, and behavior; Trash Monsters (Bunny and Gnome) [8] improves knowledge about waste separation; and Orwell: Keeping an Eye on You (Osmotic Studios) [9] raises awareness about government surveillance. In some cases, games that have not been intentionally developed for serious purposes can also achieve additional effects. For example, the Civilization (Take-Two Interactive Software) [10], Age of Empires (Microsoft Corp) [11], and Assassin’s Creed (Ubisoft) [12] series are primarily developed for entertainment purposes; however, when playing these games, players also learn about ancient organizations or the history of civilizations. Nevertheless, the question remains: what are important constituents of serious games and, in particular, what are important aspects of high-quality serious games? Although many useful heuristics are presented in the game-related literature [13,14], no model yet exists that focuses equally on the serious and game aspects, as well as on the balance between them.

Existing quality criteria for video games often focus on appropriate game design, (eg, they consider only the player enjoyment [13] and are identified based on game reviews and rating systems [14,15]). Rating systems cover the different kinds of expertise of involved game reviewers and the complexity of testing processes [16]. For example, IGN (IGN Entertainment), Gamespot (CBS Interactive), and PC Gamer (Future US Inc) primarily rely on the expertise and opinions of their in-house editors. The metareview system Metacritic (CBS Interactive) aggregates these expert scores into a single metascore, in addition to letting users vote on a secondary user score. Conversely, studies that evaluate quality criteria for serious games are often specific to an application field and focus on didactic aspects (ie, they propose specific guidelines for educational [17] or motion-based serious games [18]). In particular, existing quality criteria for serious games often lack in the combining of both aspects (ie, serious as well as game aspects).

In this paper, we aimed to gather criteria for high-quality serious games, considering both the serious and game aspects and the balance between them. First, we described some of the successful serious games according to the proven “DIN SPEC 91380 Serious Games Metadata Format” (SG-MDF) [16,19]. Existing serious games taxonomies include specific classification systems for rehabilitation games [20], educational games [21,22], or serious games in general [23-25]; however, they usually select classification criteria arbitrarily and are not generally accepted. In particular, they do not take the aspects of the characterizing goal or the quality of serious games into consideration. SG-MDF overcomes these limitations and covers all crucial aspects of serious games (ie, the characterizing goal as well as quality criteria). Finally, based on the description of serious games, relevant game-related work, and close cooperation with domain experts, we refined and extended these quality criteria to define high-quality serious games.

To summarize, our primary goal is to identify quality criteria for the serious and game part, as well as the balance between them. We have provided guidelines for high-quality serious games drawn from literature analysis and workshops with domain experts.

## State of the Art

### Game Classification and Selection

We studied serious games to classify them according to SG-MDF. SG-MDF is also used in a metadata-based information system [26], which allows providers of serious games (eg, developers and publishers) to systematically describe the games so that users (eg, teachers, trainers, coaches, doctors, and therapists) can find suitable games accordingly. Using this format, we provide a summary of games for health [3,4,6,7,27-52] and educational games [2,8,9,53-64] (see Multimedia Appendices 1 and 2). Note that the list is not complete and should only serve as a foundation for developing and explaining the quality criteria. We selected serious games that prove their effectiveness either through scientific studies or by winning game awards. Furthermore, the selected serious games have a certain level of familiarity in the community.

We use SG-MDF because it overcomes the limitations of the existing taxonomies [23-25] and covers crucial aspects of serious games, such as the characterizing goal and the quality (based on scientific studies, game awards, professional ratings, recommendation by experts, and number of players/downloads). However, we did not include all categorizations as proposed by SG-MDF and included only measures that are important to present the quality criteria in this paper. For example, game modes are important for the “support social interactions” criterion, and the target group is essential for the “appropriate interaction technology” and “media presentation” criteria. In general, all serious games should use appropriate interaction technology for the target group, game purpose, and application area. Furthermore, the progress indicator is essential for the “appropriate feedback and reward” criterion.

### Games for Health

Serious games are not only fun to play but are also beneficial for health. For example, they can motivate players to increase physical exercise. Due to insufficient physical activity, the risk of diseases such as obesity, diabetes, cancer, and cardiovascular diseases are increasing. The World Health Organization reports that physical activity has decreased over time in high-income countries [65]. These results show that it is crucial to motivate people to become more physically active. However, games for health do not only cover physical exercises but are also often used for prevention, rehabilitation and, in general, supporting health (ie, enforcing a behavior change towards a better, more active, and healthier lifestyle, including better nutrition).

Popular exergames such as Pokémon GO [27] and Dance Dance Revolution (Konami) [28] aim to provide an effective and attractive workout experience for a wide variety of users. Pokémon GO, for example, has over 1 billion downloads on Google Play Store (Google Corp) [44], making over $800 million US dollars worldwide in 2019 [45]. The study by Althoff et al [4] shows that it indeed increases players’ activity level compared with their prior activity level; however, the researchers could only confirm short-term effects. Additional studies show that Dance Dance Revolution significantly increases energy expenditure [29] and improves aerobic fitness in overweight children [30]. Some schools have even included the game in their physical education courses to motivate children to exercise [66]. However, studies also report that exergames are often only capable of providing light to moderate exercise and thus, often fail to significantly increase physical activity or exercise attendance [67].

Furthermore, Wii Sports games (Nintendo Co) are best-selling video games [68] that contribute to weight loss [31] or to increased muscle strength [32]. However, they lack proper training concepts and disregard performance aspects that are essential for a successful workout (eg, accuracy, precision, and intensity of movement). Similarly, Beat Saber (Beat Games) [33] is one of the top virtual reality games in 2019 [46,47]; however, it focuses on game design and ignores the extensive knowledge of movement and training science in sports. On the contrary, the ExerCube [34] was developed by an interdisciplinary team of sports scientists, game designers, and researchers in the field of human-computer interaction. The results of a user study with 40 participants show that the ExerCube is on par with personal training [3].

Other serious games intend to improve the physical status of older people. For example, BalanceFit aims to improve coordination, strength, and balance [35]. A study by Hardy et al [36] shows that an adaptive approach enables people with heterogeneous skills to play this game (eg, fit players as well as players with gait impairments or wheelchairs). Furthermore, the game ErgoActive provides adaptive cardio training on an ergometer bike to increase the physical activity of its players [35]. The results of a feasibility study with 16 participants demonstrated the effectiveness of cardio training based on personalized heart rate control [37].

The characterizing goal of a game for health does not necessarily need to aim at a physical training effect. The serious game Re-Mission [38] intends to inform patients about cancer treatments and aims to change their attitude positively. Studies confirmed the effectiveness of the game in randomized controlled trials with cancer patients [6,7]. Other serious games, such as Escape from Diab and Nanoswarm: Invasion from Inner Space (Archimage) are persuasive and are able to change health-related behavior among children [39]. Further serious games for health are used as prevention (eg, PlayForward: Elm City Story) [40] or rehabilitation (eg, SnowWorld) [41]. Moreover, Dr Kawashima’s Brain Training (Nintendo Co) includes a set of minigames that are designed to improve cognitive functions in elderly persons. However, even though randomized controlled trials report benefits [42,43], no long-term effects and relevance for everyday functioning could be confirmed.

### Educational Games

In addition to motivating players to become more physically active, serious games are often used to increase players’ motivation levels to learn and improve learning outcomes. Educational games can be a reliable and effective tool compared with traditional methods [69]. According to the Entertainment Software Association, 74% of parents believe that video games can be educational for their children [70]. Educational games are also the second most popular Google Play app category [71]. Serious games are effective in terms of learning and some of them are even better at teaching than traditional methods [72].

Studies in the field of game-based learning show the benefits of educational games, including improvement of mathematical skills (eg, Meister Cody – Talasia [2]), reading performance (eg, Meister Cody – Namagi [53]), and programming skills (eg, Debugger 3.16: Hack’n’run [Spiderworks Games] [54,61]). Serious games can also be used to assess knowledge (eg, Semideus [Flow Factory] [55,56]). VocabiCar (Westermann Digital GmbH) [57] is another educational game for children and intends to expand the English vocabulary of pupils.

In addition to improving players’ skills, educational games can also raise awareness. The game Trash Monsters [8] raises awareness of waste separation and teaches children how to recycle correctly. Educational games do not necessarily need to be intended for children but can be dedicated to students or adults in general. Orwell: Keeping an Eye on You serves to raise awareness of state surveillance [9,63], and Orwell: Ignorance is Strength (Osmotic Studios) serves to raise awareness of fake news [58,64]. By representing different moral values, the game strengthens or enforces players’ opinions. However, both serious games are reading intensive and therefore (similar to all serious games) only suitable for specific player types.

Another application area for serious games are simulations, in particular, corporate games for training purposes. ViPOL (TriCAT) enables police forces to train in virtual reality for scenarios that are too expensive, complex, or dangerous to be trained for in the real world [59]. The simulation was developed in close cooperation with police officers. In a study by Bertram et al [59], the results show that virtual training can be as efficient as regular training for complex collaborative tasks.

## Identifying Quality Criteria for Serious Games

### Development of the Criteria

A review of the state of the art was conducted to determine quality criteria for attractive and effective serious games. We propose essential aspects of high-quality serious games, including characteristics for the serious and game part, as well as for the balance between them. Although we focus on educational games and games for health, the quality criteria are transferable to all kinds of application areas. For example, the criteria can be used not only for games that improve players’ skills/performance but also for games that raise the players’ awareness of a certain topic or that positively change their attitudes.

**Figure 1:**
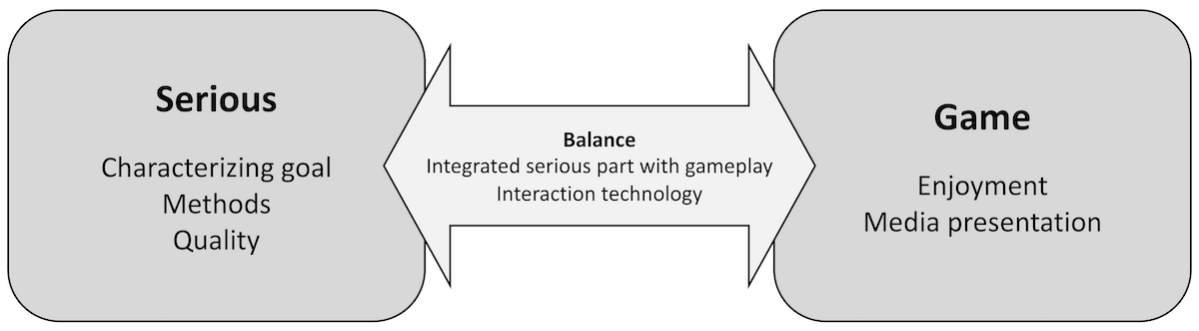
Quality criteria for the serious part and game part, as well as the balance between them.

We furthermore discussed the derived quality criteria in workshops with experts from the respective areas. The aim of the workshops was to identify the requirements and needs for high-quality serious games. Therefore, domain experts, such as game developers and companies that deploy serious games, as well as scientists from different areas (eg, sports education and computer science), critically discussed the quality criteria of serious games. The quality criteria are shown in Figure 1 and further detailed in the following section. High-quality serious games must achieve both the serious and the game aspects; they must systematically support players to reach the characterizing goal (serious part) and they must elicit and maintain player experience (game part). Furthermore, both parts should be perfectly matched and integrated rather than addressed in isolation.

In the following section, we summarize key findings, including the strength of evidence for each criterion. These results should help other researchers and game developers gain a deeper understanding of high-quality serious games.

### Serious Part

This section describes the core elements for the serious part of the game: existence of a characterizing goal, development of appropriate methods for achieving this characterizing goal, and evaluation of the quality (see also Table 1).

**Table 1:** Summary of quality criteria for the serious part.

Quality criteria and relevant quality aspects			Explanation
**Characterizing goal**			
	Focus on the characterizing goal	Learning/training goal must remain in focus, for which a combination of physical and cognitive training can be beneficial Support players to achieve the characterizing goal Game elements should not interfere with the learning/training process	
	Clear goals	Appropriate methods for the specific application area and target group Goals are clear and appropriate so that players can work towards the characterizing goal	
	Indispensability of the characterizing goal	Serious part must be mandatory Characterizing goal must not be avoidable Training and learning tasks should not be a hurdle	
**Methods**			
	Correctness of the domain expert content	Avoid errors and ensure that the content is technically correct Ensure correct technical language Remain neutral, especially on political and social issues	
	Appropriate feedback on progress	Players should receive feedback on their performance and progress Visible and recognizable effects Provide simultaneous feedback (eg, visual, audio, haptic; multimodal feedback)	
	Appropriate rewards	Provide positive reinforcement and in-game awards	
**Quality**			
	Proof of effectiveness & sustainable effects	Prove that the characterizing goal is achieved Learning/training effects need to be sustainable	
	Awards and ratings	Game awards, professional and user ratings, recommendations by domain experts, game reviews, and number of players/downloads state the quality of the game	

#### Characterizing Goal

Serious games must ensure that players achieve the characterizing goal.

##### Focus on the Characterizing Goal

The characterizing goal of a serious game is closely linked to the application area. In educational games, the characterizing goals include learning or training effects. In games for health, the characterizing goals include changes in vital status or general changes in attitude and behavior (eg, nutrition or mobility behavior). It may also be beneficial to combine physical and cognitive training. For example, in the game Lü (Lü Interactive Playground) [73], the players not only improve mathematical skills but also stay physically active. Similarly, ExerCube [34] provides training for body and mind. Furthermore, recent evidence on exergame-based therapy for Parkinson disease shows that exergames can enhance cognitive skills and are at least as effective as traditional therapies [74,75].

Thus, serious games should always focus on achieving the characterizing goal and should support the player in achieving this goal. Learning or training content must remain in focus during gameplay and game elements should not interfere with the learning or training process.

##### Clear Goals

Similar to entertainment games, in which the game goals should be clear [13,14], the characterizing goal of a serious game should also be transparent so that players can work towards achieving this goal. In particular, a serious game should ensure that players always know what to do to complete the tasks or exercises; otherwise, a tutorial is required (see also the “Intuitive Game Mechanics and Natural Mapping” section). For example, in the ExerCube, a virtual avatar demonstrates required movements in a short tutorial to make sure that players know how to execute them [3]. The educational games Meister Cody – Talasia and Meister Cody – Namagi [60] show how each exercise should be solved before players start to solve the tasks.

##### Indispensability of the Characterizing Goal

Engaging in the serious part of the game should be mandatory for playing the game. Otherwise, players may avoid the serious part to get to the fun part more quickly [76], preventing the characterizing goal from being reached. In particular, the training and learning tasks should not be a hurdle while playing a serious game. In other words, the characterizing goal should be embedded in the gameplay (see also the “Integrated Serious Part With Gameplay” section).

#### Methods

The methods for serious games need to be appropriate for the specific application area and target group.

##### Correctness of the Domain Expert Content

The most evident requirement for serious games is that they must not contain any errors with respect to their subject matter, such as erroneous mathematical equations, incorrect information on historical events, or inadequate information on physical exercise. If typing or presenting errors occur, these errors must not mislead players. Furthermore, conveyed information must not only be factually correct but also be imparted using appropriate technical language.

Moreover, even though there is usually no connection between neutrality and the correctness of a given opinion or issue, serious games should remain neutral, especially on political and social issues. For example, the games Orwell: Keeping an Eye on You [9] and Orwell: Ignorance is Strength [58] do not convey a specific political opinion to the player. Instead, they only show the effects of the player’s actions without judging them. The player then has the opportunity to assess and question their own decisions. In particular, serious games should be appropriate for the target group, depending on religion, culture, and traditions.

##### Appropriate Feedback on Progress

A serious game should provide appropriate feedback to players so that they can assess their progress. Thus, to enhance player performance, effects should be visible and recognizable (eg, through a progress bar). Continuous feedback on progress is essential in all serious games as the players work towards achieving the characterizing goal [77]. Moreover, multimodal feedback (eg, visual, audio, or haptic feedback) can be beneficial [14]. For example, in the ExerCube, players receive immediate visual and audio feedback to enhance their movements [3].

Apart from in-game feedback, Ravyse et al [78] furthermore show that postgame feedback also improves learning. Similarly, the level number can indicate the player’s progress (ie, the higher the level, the more skills the player has developed). A higher in-game level seems to have a more significant effect on motivation (desire to practice) than individual rewards (eg, achievements) [79]. Game statistics additionally show the player’s progress after ending the game or a level and are not only advantageous for players themselves, but also useful for their therapists (especially in games for health [20]) or their parents and educators (in educational games [60]).

##### Appropriate Reward

Games should provide positive reinforcement and in-game awards [1] to immerse players more deeply in the game [14]. For example, in the educational game VocabiCar, players who accomplish a learning task or complete a challenge gain points and can access their progress in a high-score table [57]. High-score tables allow players to compare their performance against other players. In addition to points, in-game awards consist of virtual badges, achievements, power-ups, and desirable objects. Fancy animations or a possibility to change their avatar (eg, new clothes, hair color, or equipment) as a reward for accomplishing a task can further motivate players. However, there is ample evidence that “rewards or feedback delivered in a controlling manner undermine intrinsic motivation and deeper forms of learning” [80]. Therefore, rewards should be deliberately deployed in serious games.

#### Quality

High-quality serious games should measure the effects and benefits in a scientific study. Furthermore, awards and user or domain expert ratings can confirm the quality of a serious game.

##### Proof of Effectiveness and Sustainable Effects

A serious game is effective when players achieve the characterizing goal and the learning or training effects are sustainable. Researchers often validate effectiveness with a study, such as a scientific, clinical, or empirical evaluation, by monitoring heart rate (eg, ExerCube [3] and ErgoActive [37]), number of steps (eg, Pokémon GO [4]), or aerobic fitness (eg, Dance Dance Revolution [30]). However, potential aversions to video games, certain game genres, or specific interaction technologies among players have to be considered when designing and evaluating a study. In educational games, the results of a group exposed to a serious game and a group exposed to traditional methods can be compared (eg, Meister Cody – Talasia [2] and ViPOL [59]). However, empirical studies often suffer from numerous sources of error (eg, bias) [81]. In this regard, randomized controlled trials with a sufficient number of participants are the gold standard for empirical proof of effects.

##### Awards and Ratings

In addition to scientific studies that evaluate usability and player experience, game awards (eg, German Computer Games Award, European Innovative Game Award, and International Educational Games Competition) are also an important aspect of identifying high-quality serious games. Further quality criteria include professional or user ratings, the number of players or downloads (eg, Google Play, App Store [Apple Inc], and Steam [Valve Corp]), and recommendations by domain experts and game reviews (eg, IGN, GameSpot, and PC Gamer).

### Game Part

This section describes core elements for appropriate game design and suitable interaction technology, as seen in Table 2. Note that there is considerable overlap in the various concepts within the game part.

**Table 2:** Summary of quality criteria for the game part.

Quality criteria and relevant quality aspects			Explanation
**Enjoyment**			
	Ensure player engagement and experience	Ensure positive experience during playing Serious games should be engaging and enjoyable (Koster’s theory of fun for game design [82], GameFlow approach [13], and PLAY^a^ heuristics [14]) Provide an engaging experience for different player types	
	Ensure flow	Keep a balance between a player’s skills and challenge (Csikszentmihalyi’s flow theory [83]) Dynamically adapt the difficulty level depending on the current player’s performance in the game Adapt to players to increase effectiveness (eg, motivate them to repeat the exercises continuously and regularly) Increase complexity as the player gets better (Bushnell’s theorem of “easy to learn, difficult to master” [84]) Provide varied gameplay	
	Establish an emotional connection	Allow emotions and arouse instinct (Dillon’s 6-11 framework [85], LeBlanc’s theory of 8 kinds of fun [86])	
	Sense of control	Players should have control over their actions in the game	
	Support social interactions	Provide different game modes (collaborative and competitive settings for players that perform better in groups)	
	Ensure immersive experience	Include multimodal sensory stimulations: visual, audio, haptics, smell Ensure the sense of “being there”	
**Media presentation**			
	Attractive graphics	Graphics must be appropriate for the game purpose, application area, and target group Ensure clear interface without unnecessary information to not distract players from a specific task	
	Appropriate sound	Include appropriate background music and sound effects	

^a^PLAY: Heuristics of Playability.

#### Enjoyment

Serious games should not only ensure positive player experience, flow, and sense of control but should also support social interaction.

##### Ensure Player Engagement and Experience

Player engagement is tightly associated with enjoyment. Koster, the author of *A Theory of Fun for Game Design*, addresses the importance of a game being engaging, enjoyable, and fun [82]. The GameFlow approach proposed by Sweetser and Wyeth [13] includes 8 dimensions with numerous criteria and recommendations to ensure player enjoyment in games. Calvillo-Gámez et al [87] furthermore present the core elements of the gaming experience to provide a positive experience while playing video games. Moreover, Desurvire and Wiberg [14] propose heuristics of playability for game developers to develop better games.

However, due to different kinds of players, not every player will find all components equally important. Therefore, the game should provide different fun components to provide an engaging experience for different player types (eg, Bartle’s player types [88]).

##### Ensure Flow

For the optimal player experience, the game has to establish a satisfying balance between challenges and skills. Csikszentmihalyi’s well-known flow theory describes the feeling of enjoyment when the task difficulty and skill levels are in balance [83]. This theory is complemented by Bushnell’s theorem of “easy to learn, difficult to master” [84]. Games that are easy to learn enable flow because they are not overwhelming, whereas games that are hard to master keep players from dropping out because of boredom. Thus, as the player’s performance improves, the complexity or difficulty of the game should also increase. In other words, serious games should adapt to the current performance level of the player (ability vs skills).

To avoid boredom, game developers should also ensure that the gameplay varies. As proposed by Desurvire and Wiberg [14], any fatigue or boredom should be minimized by varying activities and pacing during the gameplay. Furthermore, research by Scoresby and Shelton [89] identified that content, emotion, motivation, and engagement associated with the game are necessary criteria for flow.

Moreover, serious games should automatically adapt to the players to motivate them to keep learning/training and to increase the effectiveness. One of the primary advantages of educational games is their ability to engage the learner so that they voluntarily complete sufficient repetitions of activities, ensuring that learning takes place [90]. For example, both of the Meister Cody educational games adapt their difficulty depending on the player’s skills [60]. The results show that, due to adaptivity, game-based learning is particularly promising for children who want to learn in a home environment or do not have access to individual reading support [53]. Similarly, exergames should ensure that the intensity matches the player’s fitness level in order to motivate players to repeat the exercises continuously and regularly [91]. For example, ExerCube [3] or ErgoActive [37] identify the individual’s optimal strain to adapt the game difficulty and complexity gradually based on the player’s heart rate.

##### Establish an Emotional Connection

Additionally, players should get emotionally involved in a serious game. Dillon [85] has drawn attention to the fact that emotions and instincts increase players’ engagement to continue playing the game. The game designer and developer proposed the 6-11 framework, which contains 6 basic emotions and 11 instincts. For example, various serious games use an instinct to survive and thus to fight (eg, Re-Mission [38]) or to collect something (eg, VocabiCar [57] and Debugger 3.16: Hack’n’run [54]). Furthermore, LeBlanc’s theory of 8 kinds of fun describes the desirable emotional responses evoked in players when they interact with the game [86].

##### Sense of Control

Players should feel in control over their actions in the game world [14]. In particular, players should have control and influence on the game world. For example, in the serious game PlayForward: Elm City Story, players can see how different actions lead to different outcomes [48]. Similarly, Escape from Diab allows players to influence the storyline and the characters [51,52]. As a result, the ability to influence the game world and in particular the story progress can motivate players to keep playing the game. Furthermore, serious games should support an optimal relationship between the player’s actions and the game’s reactions. For example, increased pedaling frequency in ErgoActive will always cause the character to rise [35].

##### Support Social Interactions

Bond and Beale [15] identified that good games offer some form of social interaction (see also the self-determination theory [92]). Social interactions in games are important for players who perform better depending on the game mode (eg, playing with friends or against them). For example, Pokémon GO [27] lets friends feed the player’s creatures, and friends and family playing Dr Kawashima’s Brain Training [49] can compete against each other. Vorderer et al [93] suggest that competitive elements are important for enjoyment. On the contrary, the work of Staiano et al [31] reveals that playing a cooperative version of the Nintendo Wii Active game is more effective than playing a competitive version. Thus, different game modes motivate players more or less. Especially if the players perform better in groups, collaborative and competitive multiplayer settings can contribute to motivating players [3]. For specific players, playing in a group (ie, multiplayer games) is more motivating than playing alone (ie, single-player games) [86]. Depending on the game purpose and target group, the game developers and designers should try to include different game modes so that a serious game is enjoyable for a broad player base.

##### Ensure Immersive Experience

Immersion in virtual environments can be increased by stimulating different human senses, especially by including appropriate audiovisual elements in the game. The game should use visceral, audio, and visual content to immerse players more deeply in the game [14]. Slater and Wilbur [94] describe immersion as the extent to which the computer system can deliver an illusion of a virtual environment to players. Thus, an immersive virtual environment should accommodate a wide range of appropriate synchronized sensory modalities. Recent studies already provide evidence that multimodal sensory stimulation improves the sense of presence (ie, the sense of “being there”) and immersion [95]. For example, SnowWorld [50] successfully distracts players during rehabilitation (wound treatment) by immersing them in a virtual world. For a fully immersive virtual reality experience, serious games should include visual (eg, current-generation head-mounted displays), audio (eg, noise-canceling headphones), and haptic (eg, data gloves with force feedback or vibrations) feedback, as well as sense of smell (eg, smell dispenser) [96].

#### Media Presentation

One of the most apparent factors for immersive serious games is that they should have visually appealing graphics and appropriate sound effects. In particular, audiovisual elements in the game seize the attention of players [78].

##### Attractive Graphics

The included graphics should look attractive and engaging, as well as appropriate for the game purpose, application area, and the target group. For example, a game should have different designs for children, adults, and people with disabilities. The game designer should ensure clear interfaces without unnecessary information. Ravyse et al [78] furthermore suggest creating games that are high in realism; however, it should not be overloaded with unnecessary objects so that players do not get distracted from a specific task. For effective training, particularly for firefighters [97] or police training [59,98], the simulations should provide realistic virtual environments. However, in contrast to high-end graphics, reduced graphics can also be appropriate for some game types. For example, Minecraft (Mojang Studios) [99] is one of the most successful video games and it contains a world of blocks.

##### Appropriate Sound

Serious games should not only be visually appealing but should also include appropriate background music and sound effects. Previous studies have shown that audio influences the sense of presence, particularly in immersive virtual reality applications [100,101]. Martin-Niedecken et al [3] have expressed a similar view. The researchers show that music increases the motivation and immersion of the test subjects while playing the ExerCube. However, due to the players’ varying music preferences, the choice of music could also be an important factor in motivation [102]. For example, the game Beat Saber allows players to create levels with custom songs [33].

### Balance Between Serious and Game Part

The serious part and the game part of the game should be integrated and strongly connected, as seen in Table 3.

**Table 3:** Summary of quality criteria for balance between the serious and game part.

Quality criteria for balance and relevant quality aspects		Explanation
**Integrated serious part with gameplay**		
	Embedding characterizing goal into the gameplay	Integrate the characterizing goal into the gameplay Learning/training tasks must be related to the game and should be connected to the game elements
	Scientific foundation	Include interdisciplinary teams; game designer and domain experts should work together (also together with the target group) Include state of the art in the relevant disciplines
**Interaction technology**		
	Appropriate interaction technology	Interaction technology must be suitable for the target group (ie, their physical and mental ability and game purpose)
	Intuitive game mechanics and natural mapping	Provide tutorials for complex games; otherwise, players should discover the game mechanics themselves Intuitive use of game controls (eg, the *WASD* keys to move and space bar to jump) Enable natural mapping between technology and gameplay
	No simplifying of the learning and/or training process due to technical features	Interaction technology must support players in achieving the characterizing goal Ensure accurate tracking to prevent cheating in exergames
	Avoid adverse effects	Low risk of accidents, injuries, or overload Avoid technical issues and ensure easy maintenance

#### Integrated Serious Part With Gameplay

Serious games should embed the characterizing goal into the gameplay and the characterizing goal should not be avoidable.

##### Embedding Characterizing Goal Into the Gameplay

The gameplay experience includes, among other factors, an imaginative immersion (ie, immersion in the game world and the story) [95]. Thus, to motivate players, learning/training tasks need to be embedded into the immersive gameplay, such as in a story or narrative. Learning and training tasks should be directly related to the game and should be connected with the game elements and environment. For example, the educational game Addy (Coktel Vision) does not integrate learning into the game mechanics but uses a game only as a reward for learning. In this case, the serious part is not integrated into the game.

High-quality serious games should always integrate the serious part with the gameplay. For example, in adventure-based games, such as Meister Cody – Namagi and Meister Cody – Talasia, the tasks are embedded in a narrative and the story only proceeds when the player solves a problem. Similarly, Semideus [62] integrates the player’s knowledge of rational numbers seamlessly into the gameplay. This close connection between the learning/training tasks and the storytelling makes the game even more motivating for children. An engaging story can motivate players; however, games without any story can still be very successful (eg, ExerCube [34]). Therefore, a story does not necessarily have to be profound or fascinating, but depending on the type of game, it can still be motivating.

##### Scientific Foundation

To develop an effective and attractive serious game, members of an interdisciplinary team of game designers, programmers, artists, and domain experts have to work together throughout the entire development process. As proposed by Martin-Niedecken et al [3], the target group should also be involved in the design process from the beginning (ie, participatory game design). This interdisciplinary team has to establish a balance between the disciplinary standards and requirements and the interdisciplinary integration under the twofold mission of serious games. Thus, for a high-quality serious game, one needs scientific foundations on both sides [1]. However, due to the limited development budget for serious games, there are often no resources for hiring professional game designers or artists. Thus, the challenge of an optimal balance between the serious and the game parts remains, making it difficult for the teams to develop a high-quality serious game that is entertaining and also fulfills its characterizing goal. Nevertheless, teams should continue to ensure an appropriate balance.

#### Interaction Technology

Depending on the serious game and the target group, adequate interaction technology must be deliberately chosen based on the specific needs of users and the game’s purpose.

##### Appropriate Interaction Technology

The interaction technology must be suitable for the physical and mental ability of the target group. For example, the Kinect sensor (Microsoft Corp) and the Leap Motion Controller (Ultraleap) are particularly suitable for rehabilitation games, such as games for players with Parkinson disease or players who have had strokes, since these players often cannot hold or wear additional sensors [74]. Thus, depending on the target group, serious games must be presented by appropriate technology, including the appropriate visual display, speakers, and haptic interfaces, as well as suitable game-specific controllers (eg, gamepads or joysticks) [103]. The usage of an innovative technology should be appropriate for the game purpose as well; for instance, a head-mounted display can be annoying during sports, especially if the player is excessively sweating. However, commercial virtual reality games such as Beat Saber [33] have already proven that immersive environments are engaging and motivating despite sweating.

##### Intuitive Game Mechanics and Natural Mapping

It should be easy for players to understand how to play. Otherwise, the game should provide a tutorial. A tutorial should introduce not only game mechanics (how to do something) but also the gameplay (what to do). For inexperienced players, the controls should be basic enough to learn quickly, whereas experienced players can use advanced options. Desurvire and Wiberg [104] proposed principles for game designers to create better tutorials. In general, the game should be developed in such a way that players do not need to read a tutorial in order to play [14]. The work of Andersen et al [105] shows that tutorials are only appropriate for complex games and that simpler games should allow the players to discover the game mechanics themselves. Regardless of which interaction technology is used, the game controls should be intuitive, such as using the *WASD* keys to move and the space bar to jump (well-known game controls).

In addition to intuitive game mechanics, a game should also ensure natural mapping between technology and gameplay; in other words, the game should naturally map its controls to changes in the virtual environment [14]. For example, games for health should sense the movements of players to trigger the corresponding game responses [103]. Beat Saber [33] fulfills this requirement and allows players to intuitively move in the real world by simply walking around and using arm movements to slash the blocks.

##### No Simplifying of the Learning/Training Process Due to Technical Features

The interaction technology must support players in achieving the characterizing goal and must not impair or even disturb the learning/training processes. In exergames, players should not have advantages due to poor interaction technology (eg, cheating in Nintendo Wii sports games). The system must ensure that players are moving their bodies as required by the game [25]. Without accurate tracking, Wii players can sit on a couch and successfully play the game with a controller without performing the desired physical exercise. In this case, the system can be tricked and players only pretend to carry out a movement correctly. A study by Marks et al [106] shows that games played with Wii controllers require less physical activity than games played with the Kinect. To address this, exergames should track and detect motions accurately and in real time to ensure that players correctly execute all exercises. If the movement of a specific body part is required, this body part should be explicitly tracked to avoid cheating. For example, the ExerCube uses virtual reality trackers (HTC Vive; HTC Corp) to track upper body movements accurately [3].

##### Avoid Adverse Effects

Serious games should ensure that no accidents or injuries can happen, as older people can easily stumble and fall. Especially in exergames, the movements have to be tracked accurately so that players perform the exercises correctly and do not make mistakes that cause injuries. If required, the technology should be adapted and personalized to the specific target groups. For instance, the game BalanceFit uses a stability frame for secure balance training so that older people with heterogeneous skills are able to play the game [35]. Furthermore, physical or mental overload should be prevented by adequate monitoring of the player’s psychophysical state or through regular breaks suggested by the game.

Independent of the application area, all games should avoid technical issues and should be easy to maintain [15]. Moreover, in immersive virtual reality, it is important to maintain a high frame rate, low latencies, and fast synchronization to avoid cybersickness. Cybersickness is caused by perceived motion or sensory mismatch, usually reported in a virtual roller coaster or car simulations. The latency between a user’s movement and visual feedback in virtual reality has a significant impact on user experience and performance [107].

## Conclusion

In this paper, we proposed criteria for high-quality serious games. We examined various serious games and existing heuristics from game-related literature to specify quality criteria for effective and attractive serious games. The suggested quality criteria were furthermore discussed in workshops with domain experts. We introduced quality criteria for the serious and game aspects, as well as the balance between them. First, high-quality serious games should keep the characterizing goal in focus and should use appropriate methods for the specific application area and target group. Serious games should provide suitable feedback so that players can assess their progress and work towards achieving the characterizing goal. The effectiveness of serious games should be proven in scientific studies or by winning game awards. Second, high-quality serious games should be fun and enjoyable. They must ensure player engagement and should keep the players in flow (ability vs skills). Finally, the double mission of serious games, that is, the balance between the serious and the game part, must be ensured. Therefore, high-quality serious games should embed the characterizing goal into the gameplay so that engaging in the serious part is mandatory for playing the game. Furthermore, the interaction technology should be suitable for the target group and game purpose.

In future work, we want to evaluate the proposed quality criteria, weight them, and determine a score to specify high-quality serious games, thereby proposing a quality mark. We hope that the proposed criteria will encourage game designers, developers, and researchers in the future to develop more high-quality serious games.

## Appendix

Multimedia Appendix 1 Classification of serious games for health.

Multimedia Appendix 2 Classification of educational games.

## Abbreviations

SG-MDF: DIN SPEC 91380 Serious Games Metadata Format
