# Supplementary material for: Quality Criteria for Serious Games: Serious Part, Game Part, and Balance
Source: JMIR Serious Games. 2020 Jul 24;8(3):e19037. doi: 10.2196/19037 (PMC7414398; doi:10.2196/19037)
Supplement: Multimedia Appendix 1 [file games_v8i3e19037_app1.pdf]

Game	Characterizing goal	Game mode	Target user group	Interaction technology	Progress indicator	Quality
Pokémon GO	Exergame: improve physical activity level compared to the prior activity level; socialization: motivate the players to explore an urban environment (with friends)	Single-player, coop. multiplayer	Players: children, adults, family	Mobile devices	Points	Increase the average number of steps; however, the researchers could only confirm short-term effects (n=32.000, three months); over one billion downloads on Google Play Store; making over $800 million U.S. dollars worldwide (2019)
Dance Dance Revolution	Exergame: improve aerobic fitness	Single-player, comp. multiplayer	Intermediaries: trainers; players: children, adults	Dance platform	Points	Significant increase of energy expenditure (n=25, 15 minutes, one time); improvement of aerobic fitness in overweight children (n=35, 10-30 min session five times a week, 12 weeks in total)
Nintendo Wii Sports	Exergame: motivate players to work out, increase energy expenditure	Single-player, multiplayer	Players: children, adults, family	Nintendo Wii controllers	Points	Lose weight (n=54, 30-60 min sessions on school days, two weeks in total); increase muscle strength (n=40, 20 min sessions twice a week, six weeks in total)
Beat Saber	Exergame: aim at getting the players physically active	Single-player	Players: adults	Virtual reality system	Points, speed, reputation, success	One of the best virtual reality games in 2019 on Steam; more than 30.000 reviews on Steam and over 97% positive user ratings
ExerCube	Exergame: motivate players to stay physically active	Single-player, local multiplayer	Intermediaries: trainers; players: children, adults	HTC Vive Trackers, CAVE system, heart rate sensor	Points, speed	As effective as personal training (n=40, 10 min for all three conditions, one time); FIBO Innovation and Trend Award (2019, 2020)
ErgoActive	Exergame: improve cardio fitness	Single-player	Intermediaries: therapists; players: patients	Ergo-meter	Points	Adaptive approach is suitable to reach the intended individual heart rate (n=16, ten min, one time)
BalanceFit	Exergame: improve coordination, strength, and balance	Single-player	Intermediaries: therapists; players: patients	Nintendo Balance board	Points	Suitable for older people with heterogeneous skills (n=30, one year)
Re-Mission	Therapy: improve knowledge, change attitude positively	Single-player, multiplayer	Players: patients	PC, keyboard, mouse	Points	Randomized controlled trials confirm the effectiveness: improve treatment adherence and change attitude positively (n=375, one hour a week, three months)
PlayForward: Elm City Story	Prevention: HIV/AIDS prevention in young adolescents; risk behavior reduction	Single-player	Intermediaries: teachers; players: young adolescents	Tablet PC	Success	Improves attitude and increases knowledge about sexual health (n=333, six weeks, up to 16 h); International Serious Play award in the Healthcare/Medical category at the International Serious Play Conference (2013)
Dr. Kawashima's Brain Training (*Brain Age: Train Your Brain in Minutes a Day!*)	Prevention: stimulate the brain	Single-player, comp. multiplayer	Players: adults, family	Nintendo DS, Wii U, Nintendo Switch	Points	Improve cognitive functions in the elderly (n=28, four weeks, at least five days a week, 15 min pro session) and younger adults (n=32, four weeks, at least five days a week, 15 min pro session)
SnowWorld	Rehabilitation: reduce patient's physical pain during medical procedures, e.g., wound care	Single-player	Intermediaries: therapist; players: (burn) patients	Virtual reality system	Success	Distracts patients and can significantly reduce anxiety and pain (review evidence from multiple clinical and laboratory research studies)
Escape from Diab and *Nanoswarm: Invasion from Inner Space*	Persuasive: promoting health-related diet and physical activity change	Single-player	Players: children	PC, keyboard, mouse	Points, success	Increases fruit and vegetable consumption (n=133, 40 min per session, 9 sessions); Interactive Media Award (2006); Horizon Interactive Award (2007)
