# Supplementary material for: Quality Criteria for Serious Games: Serious Part, Game Part, and Balance
Source: JMIR Serious Games. 2020 Jul 24;8(3):e19037. doi: 10.2196/19037 (PMC7414398; doi:10.2196/19037)
Supplement: Multimedia Appendix 2 [file games_v8i3e19037_app2.pdf]

Game	Characterizing Goal	Game mode	Target group	Interaction technology	Progress indicator	Quality
Meister Cody – Talasia	Education: improves mathematical skills	Single-player	Intermediaries: teacher, therapist; players: pupils	Mobile devices	Success (coins)	Improves mathematical skills, in particular, arithmetical skills (n=59, 20 min sessions per day, 15 days in total); winner of the International Educational Games Competition (2014)
Meister Cody –Namagi	Education: improves reading skills	Single-player	Intermediaries: teacher, therapist; players: pupils	Mobile devices	Success (coins)	Improves reading performance (n=50, eight to eleven weeks); nomination at the German computer games award for the "best serious game" (2017)
VocabiCar	Education: expand the English vocabulary	Single-player, endless game	Intermediaries: teachers; players; children, pupils	Mobile devices	Points, high score, speed, time, success	German computer games award for "best serious game" (2018); Comenius-EduMedia-Award (2017)
Debugger 3.16: Hack' n' run	Education: learn the basic of programming	Single-player	Players: pupils	PC	Points, success	German computer game award for "best serious game" (2017); over 83% positive ratings on Steam
Semideus	Education: improve mathematical skills, e.g., fraction understanding	Single-player	Players: pupils	PC	Points	Player's performance correlates with students' math grades (n=54, 30 min, one time), (n=51, 30 min, one time)
Trash Monsters	Education: raise awareness for waste separation and teach how to recycle correctly	Single-player	Players: pupils	Mobile devices	Success, failure, points	Giga-Maus award for "best learning game" in category family (2016); sky special price (2014)
Orwell: Keeping an Eye on You	Education, state & government, politics: raise awareness of surveillance by the state	Single-player	Players: adults	PC		German computer games award for "best serious game" (2017); "Long Feature Award" at A MAZE. (2017); more than 5.000 reviews on Steam and over 92% positive user ratings
Orwell: Ignorance is Strength	Education, state & government, politics: increase fake news awareness	Single-player	Players: adults	PC		More than 700 reviews on Steam and over 80% positive user ratings
ViPOL	Training and simulation: improve tactical training for police forces	Coop. multiplayer	Intermediaries: trainers; players: vocational training	PC	Success, failure	Virtual training is as effective as regular training (n=23); Innovation award from the German Federal Institute for Vocational Education and Training (2011)
